# Die COVID-19-Pandemie als Impulssetzer für die Lehrgestaltung: Reflexionen aus Lehrenden-Perspektive zur Online-Lehre in der Medizinethik

**DOI:** 10.1007/s00481-021-00669-4

**Published:** 2021-11-03

**Authors:** Julia Perry, Ragna Ruhaas, Ruben Sakowsky

**Affiliations:** Institut für Ethik und Geschichte der Medizin, Universitätsmedizin Göttingen, Humboldtallee 36, 37073 Göttingen, Deutschland

## Einführung

Die schlagartige Umstellung des Lehrbetriebes auf Online-Lehre im Frühjahr 2020 durch den Ausbruch der COVID-19-Pandemie bedeutete für Studierende wie Lehrende am Institut für Ethik und Geschichte der Medizin der Universitätsmedizin Göttingen eine enorme Herausforderung, gab aber auch Anlass, umfänglicheren Gebrauch von digitalen Technologien zu machen.^1^ In diesem Beitrag beziehen wir uns auf unsere Erfahrungen aus drei Semestern Online-Lehre in den Fächern Medizinethik, Medizingeschichte und Medizinische Terminologie mit Studierenden unterschiedlicher Fachsemester in Kleingruppen und semesterübergreifenden Großvorlesungen. Dabei möchten wir einige methodische Ansätze vorstellen, die sich in der Praxis bewährt haben. Konkret machen wir dies an drei Herausforderungen fest, die uns Lehrenden in der Online-Lehre begegnet sind.

Die Erprobung der Online-Lehre in der Medizinethik weist weitgehend noch einen experimentellen Charakter auf. Vor einer Systematisierung der Ansätze wird es daher notwendig, zunächst Erfahrungswerte aus der Praxis zu sammeln, auszutauschen und zu evaluieren, unsere Reflexionen sollen hierzu einen Beitrag leisten.

## Übersicht: Verwendete Lern- und Kommunikationsmethoden

Bei der iterativen Gestaltung der Online-Lehre haben wir verschiedene synchrone und asynchrone Ansätze eingesetzt, die aus unserer Perspektive einen unterschiedlichen Nutzen für den Wissenstransfer und die Vernetzung aufweisen. Tab. 1 zeigt eine Übersicht der Ziele und Methoden, Tab. 2 zeigt eine Übersicht über genutzte Werkzeuge der Online-Lehre und ihren Funktionsumfang.

**Table 1:** 

Einsatzzweck	Asynchron	Synchron
*Kommunikation*	E‑Mail, Online-Foren & permanente Chats (Stud.IP)	Videosprechstunden & Live-Chats (Zoom, Stud.IP, BigBlueButton)
*Leistungsabfrage & Anwesenheitskontrolle*	Zeitgesteuerte Aufgaben: Einzel- und Gruppenarbeit (Stud.IP)	–
*Multimediale & interaktive Wissensvermittlung*	Filmausschnitte, Quiz, Online-Spiele, interaktive Lernmodule (Ilias), Probeklausuren (Ilias)	Ethische Fallbesprechungen via Zoom, BigBlueButton

**Table 2:** 

Werkzeug	Funktion	Referenz
*Stud.IP*	Teilnehmermanagement, Dateienupload, Medienwiedergabe, Foren, permanente Chats, Live-Chats, Videokonferenzen, Arbeitsaufgaben	https://www.studip.de
*Ilias*	Interaktive Lernmodule, Probeklausuren	https://www.ilias.de
*Zoom* *BigBlueButton*	Videokonferenzen, Live-Casts	https://zoom.us https://bigbluebutton.org

## Kommunikation: Verloren im digitalen Raum

Als besondere Herausforderung haben wir den Wegfall direkter und spontaner Interaktion sowohl zwischen Lehrenden und Studierenden als auch zwischen Studierenden untereinander empfunden. Zwischenfragen in der Vorlesung oder Diskussionsrunden im Seminar fallen bei einer asynchronen Lehre, wie bei Screencast-Vorlesungen oder Lernmodulen, weg, bzw. können nur zeitlich versetzt zur Lernstoffvermittlung angeboten werden. Bei synchronen Formaten – wie Fragestunden via Videokonferenz oder Live-Chat – kann das Diskussionsniveau einer Präsenz-Veranstaltung aufgrund geringerer aktiver Beteiligung oft nicht erreicht werden. Auch ist die Kommunikation unter Studierenden zum Teil gehemmt. So entfallen beispielsweise der niedrigschwellige Austausch mit dem Sitznachbarn sowie der Lerneffekt durch Anregungen und Fragen anderer Studierenden. An dieser Stelle müssen Lehrende Ansprechbarkeit signalisieren und neben E‑Mails und Videosprechstunden zusätzliche Kommunikationskanäle bedienen. Online-Foren und permanent angelegte Chaträume können hierbei eine zeitlich flexible Lösung darstellen, die Studierenden zudem ein Nachschlagewerk bereits beantworteter Fragen bieten. Für Lehrende hat die schriftliche Bearbeitung von Fragen den Vorteil, dezidierter zu antworten und ggf. unklare Sachverhalte zu recherchieren. Als weitere Lösung hat sich die Zuweisung von verbindlichen Arbeitsaufgaben an zuvor gebildete Kleingruppen bewährt. Hierdurch können Lehrende aktiv Anregungen zur kommunikativen Vernetzung unter Studierenden setzen.

## Umgang mit unterschiedlichen Bedürfnissen: Online-Formate als Chance

Eine weitere Herausforderung bestand darin, zwischen individuellen Lernbedürfnissen und individueller Eigenständigkeit der Studierenden einen geeigneten Mittelweg zu finden. Zwar bot die Umstellung auf Online-Lehre vielen Studierenden eine willkommene Möglichkeit, ihre Zeit flexibler zu gestalten und somit besser mit dem zeitintensiven Medizinstudium zu vereinbaren. Andere fühlten sich jedoch isoliert und empfanden es als große Herausforderung, eigenständig Lehrinhalte abzuarbeiten. Hier kam der Einsatz von vielfältigen Lehrformaten den Bedürfnissen der Studierenden entgegen. So konnten durch kürzere Screencast-Vorlesungen gezielt ethisch-theoretische Inhalte vermittelt werden, die in Einzel- sowie Gruppenarbeit weiterbearbeitet wurden. Dadurch konnte umgangen werden, dass einzelne Studierende zu stillen Teilnehmenden ohne aktive Beteiligung wurden. Durch die regelmäßige schriftliche Bearbeitung von Einzelaufgaben konnten wir als Lehrende uns einen besseren Überblick über den Leistungs- und Wissensstand aller Studierenden machen, ggf. Anpassungen für die nächsten Lehreinheiten vornehmen und wiederkehrende Fragen zentral klären. Studierende, die sich intensiver mit einer Thematik auseinandersetzen wollten bzw. konkrete Nachfragen hatten, konnten dazu eine freiwillige Ethiksprechstunde interaktiv und live nutzen. Darüber hinaus wurden multimediale Inhalte wie Filmausschnitte, Quiz, Online-Spiele und Beispiele für das Durchführen von ethischen Fallbesprechungen zur Verfügung gestellt. Online-Formate boten die Möglichkeit, mehr (Hinter‑)Grundwissen zu vermitteln, als es in der Präsenz-Lehre machbar ist, und damit gezielt auf die Bedürfnisse von Medizinstudierenden einzugehen, die in der Regel kein ethisch-theoretisches Vorwissen besitzen.

## Neue Wege: Wie gestalten wir die Hybrid-Lehre in der Zukunft?

Der Umstellung auf die Online-Lehre in den vergangenen Semestern folgt nun die Herausforderung, innovative Formate der digitalen Didaktik mit bewährten Ansätzen der Präsenz-Lehre in eine Hybrid-Lehre zu integrieren. Unter Studierenden besteht nachhaltig der Wunsch, Lernformate wie Screencast-Vorlesungen und Online-Lernmodule beizubehalten, die online zur freien Bearbeitung bereitstehen. Ihre Verwendung als Ergänzung zu Präsenz-Vorlesungen und Seminaren bietet sich besonders an, um den dichten Lehrstoff für Medizinstudierende zugänglicher zu gestalten, die mit geistes- und sozialwissenschaftlichen Methoden nicht oder kaum vertraut sind. Medizinethische Didaktik hat die Vermittlung interdisziplinärer Lehrinhalte zum Ziel und fordert Studierenden viel ab. Zur erfolgreichen Bearbeitung des Materials in Seminaren sind philosophische Grundlagenkenntnisse, medizinisches Fachwissen und Kenntnisse diskursiver Methoden gefragt. Ansprechende multimediale Online-Angebote können hier einen wertvollen Beitrag zur Vorbereitung leisten, um in Präsenz-Seminaren ein höheres Diskussionsniveau und damit auch einen besseren Lerneffekt zu erzielen, als dies bisher möglich war. Auch können jederzeit abrufbare Online-Inhalte der Vertiefung des Lernstoffs dienen. Kommunikationsangebote wie Online-Videosprechstunden, Foren und Live-Chats bilden weiterhin niedrigschwellige Möglichkeiten des Austauschs zwischen Lehrenden und Studierenden. Es bleibt zu betonen, dass Online-Methoden als Ergänzung, nicht aber als Ersatz für den persönlichen Austausch fungieren können. Der direkte und persönliche Austausch bleibt gerade bei der Vermittlung beispielsweise diskursiver Fähigkeiten unerlässlich.

## Ausblick

Wir plädieren dafür, Elemente der digitalen Lehre auch nach der COVID-19-Pandemie im Fach Medizinethik als Ergänzung zur Präsenz-Lehre beizubehalten. Sie bieten die Chance, medizinethisch relevante Inhalte ausführlicher für interessierte Studierende aufzubereiten und diese mit Lehrinhalten aus den klinischen Fächern interaktiv zu verbinden. So können auch außerhalb der regulären Veranstaltungen medizinethisch relevante Inhalte über die gesamte Studienzeit vermittelt werden, insbesondere anhand aktueller klinischer Fälle. Die kommenden Semester ermöglichen, den didaktischen Mehrwert zusätzlicher Online-Lehrangebote in der Medizinethik empirisch zu evaluieren und zukünftige Semester nachhaltig mitzugestalten.

## Footnotes

1 Auch dieser Text ist kollaborativ mit dem Dienst „GWDG ownCloud“ in einer Web-Office-Suite entstanden, https://www.gwdg.de/storage-services/gwdg-owncloud [Zugegriffen: 30. Sept. 2021].

